# Supplementary material for: Are carbon emissions trading and green financial instruments synergistic? -Comprehensive quantitative research based on content analysis
Source: PLoS One. 2024 Mar 7;19(3):e0298601. doi: 10.1371/journal.pone.0298601 (PMC10919601; doi:10.1371/journal.pone.0298601)
Supplement: S1 Data — (ZIP) [file pone.0298601.s001.zip › Data/地级市Z_F工作报告.zip!/║■─╧/╙└╓▌╩╨/└┤╫╘╣1⁄2╓┌║┼í╛┬φ┐╦╩2╛▌═°í┐_2013.docx]

各位代表：

现在，我代表市人民政府向大会作政府工作报告，请予审议，请各位政协委员和列席人员提出意见。

一、2012年工作回顾

2012年是本届政府依法履职的第一年，也是我市发展面临诸多困难和挑战的一年。一年来，在市委的坚强领导下，全市上下深入贯彻落实科学发展观，坚持以稳增长、促和谐为总要求，以承接产业转移示范区建设为总抓手，凝心聚力谋发展，改革创新求突破，转变作风促落实，科学赶超迈出坚实步伐，各项工作实现良好开局。

——经济总量突破千亿。地方生产总值完成1063．4亿元（预计数，下同）、增长12．5％，规模工业增加值完成215亿元、增长16％，固定资产投资完成803．9亿元、增长35．5％，社会消费品零售总额完成330．8亿元、增长16％，财政收入完成85亿元、增长20．6％。城乡居民收入达到19972元和6902元，均增长15％。经济总量跻身全省“千亿俱乐部”，综合实力迈上新的台阶。

——重大项目相继实施。今年是我市重大项目集中开工最多、推进力度最大的一年。涔天河水库扩建工程全面开工，永州人民几十年的梦想变成现实。神华永州火电项目成功落户，成为我市有史以来最大的投资项目。华盟万商红商贸物流中心、江华稀土产业园、湘江风光带等一批重大项目奠基开工，极大地提振了全市人民的信心，增强了永州发展的后劲。

——交通条件明显改善。永蓝、厦蓉高速公路建成通车，湘桂铁路扩能改造全面完工，永州机场恢复昆明航线，11个县区全部实现半小时内上高速，北五县区半小时经济圈、中心城区到南部六县两小时经济圈、南六县区域内一小时经济圈基本建成。

——“六城同创”顺利推进。紧紧围绕创建国家卫生城市、国家园林城市、国家森林城市、国家历史文化名城、全国知名文化旅游目的地城市、法治先进城市，着力推进城乡绿化、净化、亮化、美化，全面实施锦绣潇湘十大工程，加快建设生态新城，中心城区形象大为提升，宁远、江华、东安等县城面貌明显改观。

——人才引进成果丰硕。启动实施以五年引进2000名高层次人才为重点的“人才兴永”工程，全年引进高学历人才620名，“人才洼地”效应日益显现。

——民生保障全面加强。财政保障民生支出持续增加，占到财政总支出的70％以上。民生工程建设进展顺利，为民办实事任务全面完成。城乡养老、医疗保险实现全覆盖，就业和社会保障达到新水平，新型农村养老保险和城镇居民养老保险试点工作被评为全国先进。

一年来，我们重点抓了六个方面的工作：

一是着力稳增长。启动实施“工业四年倍增”计划，大力开展“企业服务年”活动，工业在困境中奋力前行。农产品加工、电子信息、生物医药等产业增势明显，零陵烟厂完成产值32亿元，为2015年实现百亿目标奠定了基础，长丰、湘纸等骨干企业负重奋进。县域工业发展加快，江华、宁远等县区规模工业增速超过20％。规模企业不断增多，全年新入统82家。园区发展有新变化，新增园区面积48平方公里，新建标准厂房160万平方米，道县、新田工业园区获批省级工业集中区。加快现代农业发展，实现粮食总产322．3万吨，完成烟叶收购73．6万担，实施水果扩园3万亩、低改27万亩，新建规模蔬菜基地18．5万亩，江永县被列为全国出口蔬菜基地示范县；养殖业实现稳步发展，100个标准化规模养殖场全部完工；品牌建设实现新突破，新增中国驰名商标2个、总数达到7个，新增国家地理标志保护产品1个、总数达到3个；农民专业合作组织不断壮大，农超对接快速发展；永州国家农业科技园建设加速推进，冷水滩区被列为国家现代农业示范区。现代物流、电子商务等新型服务业态发展较快，汽车、房地产消费稳步增长，全省公祭舜帝大典、第六届“和”文化节、中国盘王节等重大节庆活动成功举办，阳明山创建4A级旅游景区。金融创新步伐加快，交通银行永州分行挂牌营业，双牌农村信用联社改制为农村商业银行，市城建投、市交建投等融资平台建设得到加强。县域经济在稳增长中发挥主导作用，祁阳县进入全省经济强县行列，蓝山、道县被评为全省县域经济发展先进县。

二是着力上项目。积极开展“项目建设年”活动，全力以赴大抓项目、抓大项目。全年实施投资1000万元以上的重点项目820个，完成投资510亿元。交通建设三年大会战首战告捷，永蓝、厦蓉高速公路竣工通车，湘桂铁路扩能改造全面完成，国省干线和农村公路建设稳步推进，祁阳至冷水滩快速通道开工建设，省道323新田至宁远段、省道324蓝山段、京珠高速复线新田连接线、东安至广西全州公路基本建成，二广高速双牌连接线、国道207东安界牌至江华界牌井、国道322祁阳绕城公路改造等项目加紧实施，完成农村公路改造873公里、危桥改造148座，建成农村客运站2个、招呼站230个。农田水利建设扎实有效，实施耕地储备项目32个、完成建设规模9．7万亩，病险水库治理、农村饮水安全工程等年度任务全面完成。电网建设稳步推进，通讯网络不断完善。产业项目建设来势较好，福嘉有色、九兴鞋业、江华海螺水泥、湘祁电站、达福鑫光电、祥瑞科技、晶辰电子等项目竣工投产，零烟技改、长丰技改、烟草产业园、东安太阳能电站、乐福地医药等项目开工建设。

三是着力抓承接。全力开展大招商、招大商，全年实际利用外资6．2亿美元、增长34％，到位内资320亿元、增长52％；承接产业转移项目315个，其中投资亿元以上项目93个。湘南承接产业转移示范区重大项目推进大会在我市召开。对接合作步伐加快，与中外运长航、湛江港集团、中国排行榜传媒集团、新天地集团等央企名企签署战略合作协议，永州东盟办事处获批设立，对东盟出口增长226％，成效突出。积极扩大对外贸易，完成进出口总额2．3亿美元、增长20％，祁阳、蓝山被评为全省外贸进出口基地县。海关、商检永州办事处获国家批准，通关条件根本改善。

四是着力惠民生。全面落实就业创业政策，启动省级创业型城市建设，新增城镇就业5．6万人、农村劳动力转移就业5．7万人。积极推进社会保障体系建设，“五项保险”参保人数达到671万人次。大幅提高社会救助水平，城乡低保标准达到243．7元和105元，农村五保对象分散供养标准达到2000元，分别提高67．5元、34．5元和500元。巩固完善新农合制度，新农合参合率达到98．3％，蓝山县“5＋10＋100”新农合补偿模式全国推介。加快保障性住房建设，全年新增廉租住房7174套、公共租赁住房5096套，完成城市棚户区改造2600户、国有林场危旧房改造3274户、农村危房改造28100户。深入推进教育强市战略，基础教育、职业教育和高等教育实现新发展，完成124所学校校舍安全工程和82所合格学校建设，新建改建公办幼儿园20所，新增城区学位1．2万个，高考二本以上上线人数居全省第七位。科技创新取得新进步，实施省级以上科技项目43项，知识产权保护得到加强。文化体制改革全面完成，公共文化服务体系不断完善，农家书屋、乡镇综合文化站建设全面完成，社区文化活动中心、广播电视户户通工程加快推进，“三馆一站”免费开放。深化医药卫生体制改革，基层医疗卫生机构全部实施国家基本药物制度，疾病防控和食品药品监管得到加强，全年未发生重大传染病暴发疫情和重大食品药品安全事故。人口计生工作被评为全省先进。生态环境建设力度加大，绿色永州建设快速推进，省定节能减排任务全面落实。扶贫开发、移民工作成效显著，少数民族地区、边远山区、水淹区、林区发展步伐加快。竞技体育取得重大突破，2名永州籍运动员夺得伦敦奥运冠军，市体育馆竣工投用。国防教育、国防动员、国防后备力量建设继续加强，驻永部队和民兵预备役部队服务地方发展的作用不断发挥。审计、统计、人防、气象、地震、档案、对台、老龄、残疾人、史志等各项事业全面发展，工会、共青团、妇联、工商联等群团组织作用充分发挥。

五是着力促和谐。健全完善各级领导带头接访下访制度，切实加强矛盾纠纷排查化解，严格落实信访工作责任制和责任追究制，一批信访突出问题得到较好解决。着力强化社会管理，扎实推进治安防控体系建设，大力整治社会治安突出问题，始终保持对违法犯罪分子的严打高压态势，打击“两抢一盗”和“打四黑除四害”工作居全省第一。深入开展“打非治违”，不断完善事故防范体系，安全生产工作扎实有效，被评为全省先进。社区工作全面加强，办事大厅新建改建任务全面完成，办公条件明显改善，人员配备、经费保障统一规范。

六是着力强法治。严格依法行政，认真执行市人大及其常委会的决议决定，自觉接受市人大及其常委会的法律监督和工作监督，全力支持市政协履行政治协商、民主监督、参政议政职能，全年共办理人大代表建议、批评、意见211件，政协委员提案245件，办复率达到100％。深化行政审批制度改革，审批事项减少29项，审批时限缩短三分之一。加强行政程序建设，严格规范部门执法。完善政务服务体系，全面推行并联审批、全程代理、打捆收费、限时办结和园区封闭管理。全面启动“六五”普法。强力整治“三强”行为，发展环境不断优化。广泛开展“四深入两结对一测评”和“治懒惰、治散漫、治奢侈”活动，严格执行“六个一律处理”规定和“禁酒令”，严厉查处违规违纪人员，进一步促进了作风转变和工作落实。

各位代表，2012年是极不平凡的一年，我们能够取得这些成绩，实属不易，这是省委、省政府高度重视、正确领导的结果，是市委总揽全局、科学推进的结果，是市人大、市政协加强监督、全力支持的结果，是全市上下团结拼搏、奋力进取的结果。在此，我代表市人民政府，向全市广大干部群众、驻永部队、武警官兵、政法干警和社会各界人士，向所有关心支持永州建设与发展的同志们、朋友们，表示衷心的感谢，并致以崇高的敬意！

在看到成绩的同时，我们也清醒地认识到，工作中还有许多缺点和不足，发展中还有不少困难和问题，主要是：经济增速有所放缓，个别指标低于年初预期；工业经济实力偏弱，支撑发展的大项目、好项目不多；一些群众生活比较困难，改善民生任务仍然繁重；少数单位公信力、执行力不强，办事效率低；个别工作人员服务意识差，执法水平低，影响了经济发展环境；各类历史矛盾与现实问题交织，信访维稳压力大。对此，我们一定高度重视，切实加以解决。

二、2013年工作部署

2013年是全面贯彻落实十八大精神的开局之年，是深入实施“十二五”规划的关键一年，是为全面建成小康社会奠定坚实基础的重要一年。我们必须清醒地认识到，做好全年工作，面临的困难与不利影响：国际经济形势依然错综复杂、充满变数，世界经济已由危机前的快速发展期进入深度转型调整期；国内发展仍面临不少风险和挑战，不平衡、不协调、不可持续问题依然突出；我市部分企业生产经营困难，消费需求增长不快，加快发展的基础还不牢固。我们更要看到，加快发展步伐，面临的机遇和有利条件：党的十八大胜利召开，为我市科学赶超、开放崛起注入了强大的精神驱动力；省委、省政府及省直部门全力支持永州，为我市推进示范区建设增添了强劲的发展推动力；近年来我市经济社会发展势头良好，基础设施条件明显改善，县域经济快速发展，人民群众向心力、凝聚力不断增强，为全面建成小康社会提供了坚实的基础支撑力。全市上下一定要坚定信心、迎难而进，抢抓机遇、乘势而上，力创新优势，再铸新辉煌。

工作总体要求是：全面贯彻落实十八大精神，按照“四化两型”、“两个加快、两个率先”的战略部署，以国家级承接产业转移示范区建设为总抓手，以稳中求进为总基调，以转方式调结构为主线，以提高经济增长质量和效益为中心，进一步突出上项目打基础、抓承接拓对接、惠民生保稳定、转作风促落实，统筹推进经济建设、政治建设、文化建设、社会建设以及生态文明建设，向着全面建成小康社会的目标奋进。

发展预期目标是：地方生产总值增长11．5％，规模工业增加值增长16％，固定资产投资增长30％，社会消费品零售总额增长16％，财政总收入增长15％，城乡居民收入增长13％，进出口总额增长35％，力争全年固定资产投资突破1000亿元，财政总收入实现100亿元。城镇化率提高3个百分点。人口自然增长率控制在7．39‰以内。节能减排完成省定目标任务。

围绕上述思路和目标，切实抓好四个方面的工作：

（一）突出工业主导，培育现代产业体系

始终坚持兴工强市战略不动摇，强力推进新型工业化，大力实施农业产业化，积极发展现代服务业，着力构建现代产业体系，全面提升发展层次水平。

壮大新型工业。始终坚持大抓工业、抓大工业，深入推进“工业四年倍增”计划，集中全力打好工业发展翻身仗。突出项目兴工。全年新引进投资3000万元以上项目100个，新开工投资3000万元以上项目100个，全力支持零烟技改、长丰汽车技改、烟草产业园、江华稀土产业园、零陵百亿锰产业园等重大项目建设。加快转型升级。制定实施主导产业发展规划，以做大龙头、培育品牌为重点，加快发展战略性新兴产业，整合提升传统优势产业，重点抓好先进装备制造、电子信息、新能源新材料、农产品精深加工、矿产品精深加工、生物医药、轻纺制鞋等七大主导产业；加大扶持力度，推动骨干企业发展，零陵烟厂完成技改扩能，实现产值40亿元，长丰猎豹完成内部重组，拓宽产品市场，实现产值23亿元，湘纸加大工作力度，实现产值10亿元；落实小微企业发展政策，推动全民创业。年内新入统规模工业企业100家，新增年产值过亿元企业40家。推进“两化融合”。以建设“数字永州”为契机，促进新型工业化、信息化融合发展。加快凤凰园经济开发区和道县工业园省级“两化融合”试验区建设，深入实施“数字企业”示范工程和企业信息化“登高计划”，提升企业信息化水平。帮扶企业解困。要把发展工业摆到突出位置，强化目标管理，严格奖罚兑现。继续开展“企业服务年”活动，对每个骨干企业都要派出工作组，进行帮扶指导。对冶炼、建材、造纸等困难较大行业，制定扶持政策，落实解困措施。积极开展企业产品促销活动，引导鼓励党政机关、社会团体、企事业单位优先采购本市产品。

发展现代农业。着眼农业增效、农民增收，转变农业发展方式，大力发展现代农业。优化农业结构。加强优质稻、优质烟、优质水果、优质蔬菜、优质茶叶、富硒产业基地建设，完成高档优质稻扩面10万亩、烤烟种植30万亩、水果扩园8万亩、外销蔬菜基地建设16万亩，积极创建全国油茶示范市。着力推进标准化健康养殖，争创10个省部级畜禽水产养殖标准化示范场。提升加工水平。以实施粮油、果蔬两大百亿产业工程为重点，推进农产品加工振兴计划，年内新增4家销售收入过亿元加工企业，争创3个省级以上名牌农产品。坚持政府引导、市场运作，推动大米、油茶、竹木、果蔬等行业资源整合，提升我市农产品的市场占有率和品牌影响力。推进农业示范。加快永州国家农业科技园建设，年内完成农产品加工物流园、污水处理厂等项目建设，新引进10个以上重大项目，启动实施万亩高标准油茶示范基地建设，完成万亩蔬菜示范基地建设。推进农业现代化示范县区创建工程，每个县区发展1个现代农业示范区。加强水利建设。加快涔天河水库扩建工程建设进度，全力做好移民安置工作。坚持建管并重，抓紧实施201座小二型病险水库除险加固、10个中小河流治理和“四水治理”项目，加强小农水重点县的争取和项目建设，加大中小型水库渠系配套整治力度，加快推进金陵水库水源连通工程建设，争取金钩挂水库、芦江水库立项开工。改造中低产田11．8万亩，建设高标准农田5．5万亩。强化“三农”服务。创新体制机制，提升组织化程度。开展农民实用技术培训和农村劳动力转移就业培训10万人次，推广先进适用农机具5万台套，新发展农民专业合作社100家，每个县区开展1家农村土地信托流转试点。抓好防汛抗旱、森林防火和重大动物疫病防控工作。

搞活第三产业。促进文化旅游业融合发展。大力实施文化强市、旅游强市战略，按照文化旅游发展“双十工程”建设要求，重点抓好零陵古城、九嶷山两个核心旅游区建设，积极推进阳明山、舜皇山、浯溪碑林、濂溪文化景区、江永女书园、江华瑶族文化园、永州植物博览园、东山景区、周家大院、柳子街等景点景区建设，大力发展乡村旅游、森林旅游和休闲观光农业。加快景区提质扩容，完成柳子景区创4A，力争舜帝陵景区创5A，年内建成四星标准以上宾馆4家。加强旅游宣传促销，积极申办中国湖南国际旅游节，大力开展“千元游永州”活动，启动女书申报世界非物质文化遗产工作。促进商贸物流业加快发展。重点抓好华盟万商红商贸物流中心、兴农物流中心、市再生资源大市场、华天城等项目建设，抓好国家级农产品现代流通综合试点城市工作，推进“新网工程”、“网上供销社”建设。大力发展科技服务、信息咨询等生产性服务业，休闲健身、家政物业等生活性服务业，电子商务、数字传媒等新兴服务业。促进房地产业健康发展。认真落实促进房地产业发展的各项政策，加强普通商品房供应，鼓励普通商品房消费。推广生态节能环保型建筑，促进住房建设由数量型、粗放型向绿色环保宜居型转变，提升人居环境质量。加强房地产市场监管，规范房地产开发经营行为。集中开展小区专项整治，完善服务设施配套，注重提高品位和档次。

（二）深化改革开放，加快推进示范区建设

改革开放是促进永州赶超崛起的强大动力，必须进一步深化改革、扩大开放，加快构建有利于示范区建设和开放型经济发展的体制机制。

做强县域经济。全面落实促进县域经济发展的政策措施，实施发展特色县域经济强县工程，着力提升县域经济综合实力。积极引导各地利用资源条件，发挥自身优势，培育发展特色产业、特色园区。打破行政区域界限，强化县际区域协作，实现资源优势互补、产业错位发展。大力扶持民营经济发展，放宽市场准入，鼓励充分竞争，凡是政策和法律没有禁止的领域和行业，都允许民营资本进入。加大政策支持，完善社会服务，切实保障民营企业的合法权益。

加快承接对接。全年实际利用外资8亿美元、内联引资430亿元，新承接产业转移项目400个以上，每个县区新落户央企省企名企5家以上。推进产业链招商。围绕全市七大主导产业，科学编制招商规划，创新招商方式，着力引进一批行业龙头企业、产业领军企业和关键链条节点骨干企业。完善以商招商，扶持已落户企业壮大规模，形成倍增效应。加强对接合作。充分利用资源条件，开展与名企对接，争取一批国内500强和上市公司进驻永州。着力增进与东盟国家的投资合作，积极推动我市机械制造、食品加工、化工冶炼等企业产品进入东盟市场，鼓励有实力的企业参与东盟国家的基础设施建设和资源开发。组织参加第十届中国—东盟博览会等重要经贸活动，力争建设永州—东盟合作交流平台。扩大对外贸易。加大扶持力度，做大做强湘威鞋业、凯盛鞋业、果秀食品、奔腾彩印、恒远发电、科力尔电机等出口龙头企业，每个县区新发展2家有出口实绩的外贸企业。加大国际市场开拓力度，扩大食品、机电、纺织、制药等优势产品出口。

夯实基础平台。加快交通建设。深度推进交通建设三年大会战，力争开工建设衡道高速公路，促进湘桂铁路动车组早日开通，永州机场新开辟到发达地区、旅游景区航线。加强沟通协调，尽快实现永州与广东、广西高速公路对接互通。加快推进干线公路建设改造，全面完成永州大道城区段、省道217冷水滩城区段、二广高速双牌连接线、省道325道县祥霖铺至江永龙虎关、国道322祁阳绕城公路、国道207道县祥霖铺至江华界牌井、国道207东安界牌至零陵黄田铺改造任务，加快祁阳至冷水滩快速通道、国道207双牌大山段实施进度，开工建设省道231祁阳木梓圩至金洞、新田邬下至金鸡岭、省道328东安芦洪市至东安小江口、省道355江华分水岭至江华白芒营、省道236宁远中和至县城、省道239江永潇浦至广西灌阳边界、国道322黄母桥至枣木铺公路，力争开工零陵至双牌一级公路。启动实施永州汽车总站搬迁工程，争取新建农村公路650公里。抓紧做好永郴高速公路、桂永郴铁路、永州机场搬迁、湘桂运河、湘江千吨级航道等项目的前期工作。加快园区建设。全年完成园区扩园50平方公里，新建标准厂房200万平方米以上，新入园项目150个以上，凤凰园经济开发区申报国家经开区取得重大进展，祁阳、江华、零陵工业园建成百亿园区，双牌、江永工业园创建省级工业园。加快能源建设。全力推进神华永州火电项目建设，加快江华、江永、蓝山、双牌风电项目实施进度。抓紧建成五里牌、晒北滩等一批骨干水电项目。抓好1座220千伏、2座110千伏、11座35千伏变电站的新建续建，加快实施农网改造升级工程。加快通关建设。抓好永州港建设，做好长沙海关和湖南出入境检验检疫局永州办事处组建工作，推进公路、铁路、航空口岸和电子口岸建设。

推进各项改革。切实抓好国企改革，对28家尚未改制的国有企业，逐个制定改革方案，组建专门班子，力争年内改制到位。全面推进水利改革，确保完成年度改革任务。深入实施农村综合配套改革，创新农业经营机制，搞活农村土地流转。严格耕地保护，加强国土资源管理。加快推进医药卫生体制和公立医院改革，大力发展非公立医疗机构。切实加强财税工作，大力扶持企业发展，促进重大项目建设，不断夯实财源经济基础；全面推行信息化管税措施，从严控制税费减免缓审批，强化财政收入目标绩效考核，促进收入持续较快增长。厉行节约，严格控制一般性支出。

强化金融创新。加大信贷投入力度，继续实行信贷投入目标管理责任制，全年新增贷款100亿元。完善金融体系建设，华融湘江银行永州分行、蓝山农商行挂牌营业，中心城区农商行、东安、祁阳、江永、宁远、道县农商行改制尽快完成。新组建2家村镇银行，新引进2家股份制商业银行，实现小额贷款公司县区全覆盖。加大对民间投融资的政策支持，新增2家融资性担保公司，发起设立2家私募股权基金，确保市城建投债券发行成功。加强社会诚信体系建设，严厉打击非法集资活动，优化金融生态环境，创立省级金融安全区1个。提高住房公积金使用效率。

（三）推进城乡一体化，全力建设锦绣潇湘

坚持以中心城市建设为龙头，以推进“六城同创”和锦绣潇湘十大工程为重点，以提高城镇发展质量为核心，着力推进城乡规划、基础设施、公共服务一体化，有序推进农业转移人口市民化，促进城乡统筹协调、共同繁荣，共建共享美丽永州、幸福家园。

建设靓丽城镇。强化规划调控引领作用，全面完成中心城区控制性详规和专项规划编制，实现县城、中心镇控规全覆盖。积极推进冷零祁城市群建设，突出抓好生态新城建设，着力实施一批联城重大项目。加强中心城区市政设施配套，突出抓好湘江风光带、永州大道景观带、市民中心、冷水滩河东城市综合体、城南大道、城南大桥、河西防洪工程、纳污管网、零陵中路人防工程等建设，加快安置小区建设和棚户区、背街小巷改造，新建一批市政设施，启动实施黄标车治理。加快“六城同创”步伐，实施“十项硬件”建设，深化各项创建活动，主要创建指标达到国家规定标准，力争2014年创建成功。切实加强城市管理，突出强化控违拆违工作，全面开展非法营运车辆专项整治，启动实施数字城管。深入推进县城“五个一工程”，每个县区集中建成两个示范镇、一批标准化城镇，着重抓好路网管网配套、污水和生活垃圾处理设施、公园绿地、花卉苗木和文体娱乐设施建设，全面开展卫生城镇、文明城镇创建活动。大力表彰“十佳乡镇”，对“五差乡镇”党政班子主要负责人予以免职。

建设整洁乡村。继续抓好示范建设工程、乡村风貌工程，新启动200个重点示范村建设。加强村镇规划管理，逐步实现规划管理由城市到乡村的全覆盖。制定出台农村建房管理办法，尽快启动农村空心房改造。推进农村环境综合整治工程，零陵区要全面铺开，其它县区选择1－3个乡镇实行全覆盖推进。建立乡镇、村庄清扫保洁机制，加大清扫设施建设投入，做到垃圾入池、专人保洁、及时清运、集中处理。大力加强农村改水、改厕、改圈工作。开展环境“十佳村”、“五差村”评定活动，严格兑现奖罚。

建设秀美山川。坚持节约资源、保护环境，着力推进绿色发展、循环发展、低碳发展、可持续发展。大力开展绿化工作，完成植树造林70万亩、封山育林50万亩，完成中心城区20万株大苗进城。加强资源利用全过程节约管理，推进节能节水节地节材，降低能源、水、土地消耗强度。强化节能减排工作，抓好工业、建筑、交通运输等重点领域节能减排，加强公共机构节能，加快城镇污水垃圾集中处理设施建设，全面实施排污权交易制度。继续推进水土流失治理、水源保护、矿山整治等生态环保工程建设，强化生态脆弱地区的生态保护和修复，给子孙后代留下天蓝、地绿、水净的美好家园。

（四）坚持民生优先，促进社会和谐进步

始终坚持以人为本，切实强化民生保障，加快发展社会事业，在学有所教、劳有所得、病有所医、老有所养、住有所居上取得更大进展。

统筹发展社会事业。优化教育规划布局，推动义务教育均衡发展，精心办好永州四中、永州一中等11所省示范高中，扶持湖南科技学院发展，全面提升民办教育水平；加快永州职业技术学院、潇湘技师学院和中职学校、县级职教中心建设，促进职业教育与示范区建设良性互动、协调发展；加强教育管理和教师队伍建设，引进培育一批名校长、名教师。加快科技创新，实施省级以上科技项目40项，授权专利400件，新认定高新技术企业5家以上。加快文化事业和文化产业发展，启动市文化艺术中心、数字传媒大厦、市图书馆、市博物馆、市妇女儿童活动中心建设，继续推进广播电视户户通、农村电影放映和“三下乡”、“四进社区”等文化惠民工程，繁荣发展新闻出版、广播影视、文学艺术事业，加强网络社会和文化市场管理。加快市妇幼保健院、市儿童医疗服务体系等项目建设，完成73个新增中央投资卫生建设项目，完善基本药物制度，全面推广蓝山新农合补偿模式，促进基本公共卫生服务均等化。抓好市县福利中心、市军供站、县区救助管理站、光荣院和农村敬老院建设。坚持人口计生基本国策，促进人口长期均衡发展。新建一批城乡体育设施，积极备战第十二届全运会。继续抓好“人才兴永”工程，新引进高学历人才400名以上，切实用好现有人才。加强妇女、儿童工作。积极发展老龄事业和慈善事业。健全完善残疾人社保和服务体系。高度关心留守儿童、空巢老人和失独家庭。加大民族政策落实力度，促进民族地区加快发展。强化全民国防教育，积极探索完善军民融合式发展路子，大力支持国防和驻永部队建设，切实加强民兵工作，不断提高国防意识和国防后备力量建设水平，巩固和发展军政军民团结。统计、审计、物价、工商、法制、宗教、史志、档案等工作都要取得新的进步。

大力创新社会管理。改进政府提供公共服务方式，增强城乡社区服务功能，扩大农村社区建设试点，加快乡镇便民服务中心建设。加强应急管理和防灾减灾体系建设，提高应急处突、事故救援和抗灾救灾能力。建立健全重大决策社会稳定风险评估机制。完善和创新流动人口和特殊人群管理服务。畅通和规范群众诉求表达、利益协调、权益保障渠道，严格落实信访责任制，切实把矛盾化解在基层、消除在萌芽状态。深入开展安全隐患专项整治，夯实企业安全生产基础，坚决遏制重特大安全事故发生。深化平安永州建设，强化司法保障，加强政法队伍建设，落实阳光警务和智慧公安建设各项措施，全面加强立体化社会治安防控体系建设，严厉打击暴力恐怖犯罪、严重刑事犯罪、“两抢一盗”等多发性侵财犯罪，切实提高人民群众的安全感和满意度。

推进十项民生保障。一是推进就业保障，完成职业技能培训12万人，新增城镇就业5万人、农村劳动力转移就业5．5万人，城镇零就业家庭实现动态清零，高校毕业生就业率稳定在85％以上，新增市场主体1万个以上。在全社会营造“干部创事业、能人创企业、百姓创家业”的浓厚氛围。二是推进社会保障，五项保险参保人数达到672万人次，城乡居民医疗保险财政补助提高到280元，新农合参合率稳定在98％以上，稳步提高城乡低保、优待抚恤、五保供养和孤儿救助水平。三是推进住房保障，新建廉租住房2290套、公共租赁住房6848套，城市棚户区改造2472户，国有林场危旧房改造2651户，农村危房改造1万户以上。四是推进教育保障，加大校车投入和安全管理，建设合格学校60所，新增城区学位1．2万个，新建扩建公办幼儿园20所，新增农村教师周转房1000套。五是推进出行保障，中心城区新投入公交车辆150台，新增公交线路4条，新投入出租车200台，加强农村公路维护管理，逐步推进农村客运公交化。在生态新城开展立体交通枢纽建设。六是推进市场保障，启动农贸市场标准化建设，年内新建9家、改造29家。七是推进扶贫保障，减少贫困人口13万。八是推进服务保障，新建农超对接直销网点200个。九是推进安全保障，全面落实安全教育目标责任，强化食品药品安全监管工作，深入推进“计量六进”活动。十是推进饮水保障，大力建设农村饮水安全工程，新解决20万农村人口饮水安全问题。

三、建设人民满意政府，提升科学发展能力

面对赶超崛起的历史责任，面对人民群众的迫切期待，各级政府及其工作人员一定要以提高执行力为核心，切实强化效能建设，提升服务发展能力，努力建设人民满意政府。

牢记宗旨，服务人民。始终坚持把人民群众对幸福生活的向往作为我们矢志不渝的奋斗目标，把人民群众的信任与重托作为我们履行使命的强大动力，全心全意、尽职尽责为全市人民谋利益、增福祉。扎实开展“四深入两结对一测评”活动，深入基层、深入一线，倾听群众呼声，广交市民朋友，切实为人民群众办实事、做好事、解难事。加强“市长信箱”、“12345”社会求助热线、网络论坛等平台建设，切实做到问政于民、问需于民、问计于民。

科学决策，依法行政。自觉接受市人大及其常委会的法律监督、工作监督和市政协的民主监督，优质高效办理人大代表议案、建议和政协委员提案，认真听取各民主党派、工商联、人民团体和社会各界的意见建议。坚持科学民主决策，完善重大决策程序、重大事项听证、风险评估、廉洁评估机制，提高决策科学化水平。大力推进政务公开、政府信息公开和网上政务服务应用，加快电子政务建设，办好政府网站，健全政府发言人制度，切实保障人民群众的知情权、参与权、表达权和监督权。抓好“六五”普法，提高全民法律素质。始终坚持依法行政，着力创建法治先进城市，努力建设法治政府。严格依法办事，规范、公正、文明执法。

勇于担责，真抓实干。要忠于职守、勤勉工作，始终把心思放在谋发展上，把精力花在干事业上，把功夫用在抓落实上。要敢于负责、勇于担当，对看准了的事情、决定了的事项，要一抓到底、务求实效，对有利于人民群众的事、有利于永州发展的事，要积极探索、敢作敢为。要求真务实、真抓实干，着力转变文风会风，大力精简会议文件，切实减少迎来送往，严格控制检查评比，不断提高行政效能。完善政府绩效考核，加大督查督办力度，严厉查处不作为、慢作为、乱作为，确保政令畅通，确保推进有力，确保工作落实。

严明纪律，清正廉洁。坚定不移推进反腐倡廉工作，严格执行党风廉政建设各项规定，严厉查处各类违法违纪案件，切实做到干部清正、政府清廉、政治清明。强化政府内部层级监督和监察、财政、审计、社会监督，加快建立公共资源交易平台，加强对土地使用权出让、建设工程招投标、政府采购、产权交易、资金使用等重点领域廉政风险预警防控，从源头上预防腐败发生。力戒奢靡之风，严禁铺张浪费，把财力物力用在促发展、保民生上。

各位代表，全市上下加快实现科学赶超、开放崛起、富民强市，责任重大、使命光荣。让我们紧密团结在以习近平同志为总书记的党中央周围，在省委、省政府和市委的坚强领导下，同心同德，奋发进取，为建设锦绣潇湘、和美永州而努力奋斗！
